# The Effect of Bilateral Non-Invasive Vagus Nerve Stimulation on Stress Biomarkers, Depression, Anxiety, and Sleep in Healthy Adults

**DOI:** 10.1192/j.eurpsy.2026.11663

**Published:** 2026-07-31

**Authors:** J. Pukenaite, N. Burokiene, D. Karciauskaite

**Affiliations:** 1 Pulsetto; 2 Santaros Clinic, Vilnius, Lithuania

## Abstract

**Introduction:**

Stimulation of the vagus nerve influences biomarkers associated with both the hypothalamic–pituitary–adrenal (HPA) axis and the autonomic nervous system. The goal of the study was to evaluate the effect of transcutaneous vagus nerve stimulation (tVNS) on stress biomarkers and related symptoms and compare the effect of unilateral and bilateral stimulation.

**Objectives:**

Stimulation of the vagus nerve modulates the hypothalamic–pituitary–adrenal (HPA) axis and autonomic nervous system, influencing stress physiology and mental health. This study evaluated the safety and effectiveness of a non-invasive vagus nerve stimulation (nVNS) device (Pulsetto) in reducing stress-related biomarkers and improving self-reported symptoms in healthy adults.

**Methods:**

A total of 40 participants (21–64 years; 8 men, 29 women) were enrolled; 37 completed the study. Participants applied transcutaneous vagus nerve stimulation twice daily (8 minutes/session) for 4 weeks. Stimulation was randomized to unilateral or bilateral. Hair samples were collected at baseline and 4 weeks for cortisol and cortisone analysis. Validated questionnaires (PHQ-9, GAD-7, PSQI) assessed depression, anxiety, and sleep at baseline, 2 weeks, and 4 weeks.

**Results:**

Bilateral stimulation significantly reduced hair cortisol (p = 0.024), while unilateral did not (p = 0.159). A trend-level reduction in cortisone was observed overall (p = 0.059), with stronger effects in the bilateral group. Cortisol and cortisone changes correlated positively (ρ = 0.50, p = 0.002). Self-reported symptoms improved significantly: depression (PHQ-9, p < 0.001), anxiety (GAD-7, p < 0.001), and sleep quality (PSQI, p < 0.001), with consistent effects in both unilateral and bilateral subgroups. Overall, depressive symptoms dropped by 56%, anxiety by 45%, and sleep disturbances by 41% within 4 weeks.

**Conclusions:**

Bilateral non-invasive vagus nerve stimulation effectively reduces biological stress markers andimproves symptoms of depression, anxiety, and sleep disturbance in healthy adults. These findings highlight nVNS as a promising non-pharmacological intervention for stress-related conditions.

**Disclosure of Interest:**

None Declared

